# A sensitive, aqueous-based spectrofluorimetric approach for the determination of favipiravir in presence of its acid-induced degradation product

**DOI:** 10.1186/s13065-026-01724-1

**Published:** 2026-02-10

**Authors:** Mai H. Abd El-Fattah, Yasmine A. Sharaf, Heba M. El-Sayed, Said A. Hassan

**Affiliations:** 1 Pharmaceutical Analytical Chemistry Department, College of Pharmaceutical Sciences and Drug Manufacturing, Misr University for Science and Technology, 6th of October City, Giza, 12566 Egypt; 2 Analytical Chemistry Department, Faculty of Pharmacy, Zagazig University, Zagazig, 44519 Egypt; 3 Pharmaceutical Analytical Chemistry Department, Faculty of Pharmacy, Cairo University, Cairo, 11562 Egypt

**Keywords:** COVID-19, Eco-friendly, Favipiravir, Spectrofluorimetry, Stability indicating, Sustainability

## Abstract

The COVID-19 pandemic has emphasized the critical need for novel therapeutic approaches. Favipiravir (FAV), an antiviral drug primarily used for influenza, has shown promising potential in treating COVID-19 and other RNA viral infections. A precise, reliable, and rapid fluorimetric method was established for the quantification of FAV in pharmaceutical formulations, even in the presence of its acid-induced degradation product. The acid-induced degradation product (ADP) of FAV was prepared through forced degradation, followed by characterization using IR and MS. The method leveraged the intrinsic fluorescence characteristics of FAV, exhibiting a linear response within the concentration range of 5–80 ng/mL at 416.5 nm using the first-order derivative processing. Key methodological parameters were optimized to enhance sensitivity, achieving detection and quantification limits of 1.6 ng/mL and 4.8 ng/mL, respectively. All calibration and fluorimetric measurement steps were performed in distilled water without the use of organic solvents or buffers, making the analytical determination phase entirely aqueous and environmentally benign. This method was effectively applied to FAV in both pure drug and pharmaceutical dosage forms. Compared with previously reported fluorimetric methods, it offers the unique combination of aqueous-based operation, stability-indicating capability, and superior analytical performance. Additionally, its environmental sustainability was evaluated using GAPI, AGREE, and RGB12 metrics, which confirmed its green and eco-friendly attributes.

**Supplementary Information:**

The online version contains supplementary material available at 10.1186/s13065-026-01724-1.

## Introduction

The rapid global spread of Severe Acute Respiratory Syndrome Coronavirus 2 (SARS-CoV-2) necessitated urgent efforts in antiviral drug development and repurposing [1]. Among the therapeutic candidates investigated for the management of COVID-19, Favipiravir (FAV), a synthetic purine nucleoside analog, emerged as a promising candidate. It undergoes intracellular phosphoribosylation to form its active metabolite, Favipiravir ribofuranosyl-5′-triphosphate (FAV-RTP), which inhibits RNA-dependent RNA polymerase (RdRp) [2]. Initially developed for the treatment of influenza and investigated for Ebola, FAV has demonstrated promising antiviral activity against SARS-CoV-2. Clinical studies have reported faster recovery in COVID-19 patients, evidenced by improved radiological findings, reduced fever duration, alleviation of cough symptoms, and faster viral clearance compared to other antivirals. Consequently, FAV has been integrated into COVID-19 treatment protocols and adopted as a preferred therapeutic option in several countries [2].

Despite its clinical promise, the therapeutic effectiveness of FAV remains limited by its low oral bioavailability, attributed to variable gastrointestinal absorption and first-pass metabolism [3]. Consequently, alternative dosage forms, such as oral solutions for pediatric use and inhalable formulations for targeted pulmonary delivery, have been proposed to overcome these challenges [4, 5]. While these dosage forms are still under investigation, they represent an important step toward optimizing FAV’s pharmacokinetics and patient accessibility.

The advancement of such aqueous-based formulations necessitates rigorous stability evaluation. Given the susceptibility of FAV to hydrolytic degradation, particularly due to its amide moiety, understanding its degradation behavior in solution is essential. A drug stability study evaluates how a pharmaceutical product’s quality evolves over time under various environmental conditions such as temperature, humidity, and light. It plays a vital role in confirming the drug’s stability and is a core regulatory requirement. These studies determine the product’s shelf life, optimal storage conditions, and expiration date, ensuring the drug maintains its safety, efficacy, and quality throughout its intended lifespan. Forced degradation, particularly hydrolysis, which represents the primary degradation pathway for many biodegradable compounds, is a key preliminary step in the development of stability-indicating methods [6]. This process enables the identification, characterization, and quantification of degradation products and potential impurities. Such impurities may arise during synthesis, formulation, storage, or degradation and must be carefully monitored, as they can significantly affect the drug’s performance and safety profile [7].

While chromatographic, spectroscopic, and electrochemical techniques have been extensively used for stability-indicating analysis, chromatography remains the most prevalent due to its high performance [8–11]. However, it poses limitations such as high cost, complex instrumentation, lengthy procedures, and reliance on large volumes of organic solvents, raising environmental concerns [12–14]. In light of the shift toward greener analytical practices, spectroscopic methods have gained attention as cost-effective and simpler alternatives for routine QC [15–17]. Among them, fluorescence spectroscopy stands out for its exceptional sensitivity, minimal sample preparation, ease of use, and reduced environmental impact [18, 19].

The literature reveals several analytical methods for determining FAV, including spectrophotometric assays [20, 21], spectrofluorimetry [22–27], electrochemical techniques [28, 29], and chromatographic approaches [30–38]. Chromatographic methods offer stability assessment but lack eco-friendly attributes due to their high consumption of organic solvents, and some further depend on costly instrumentation (LC–MS) that is rarely available in routine quality-control (QC) laboratories.Reported spectrofluorimetric procedures have mainly quantified FAV either alone or in combination with other drugs, yet none were designed to be stability-indicating. Moreover, they relied on fluorescence measurements in ethanolic or buffered media—representing only a partially green strategy compared with chlorinated solvents. Nevertheless, these methods remain dependent on organic modifiers and controlled pH conditions, which limit their overall greenness. To date, no aqueous-based spectrofluorimetric method capable of quantifying FAV in the presence of its degradation products has been reported.

This study aims to propose a simple, sensitive, and sustainable spectrofluoremetric method for the determination of FAV in presence of its ADP both in bulk and pharmaceutical dosage forms. The proposed method is designed to function as a robust stability-indicating assay, capable of detecting and quantifying degradation under hydrolytic stress conditions. This is particularly relevant to support the development, quality control, and regulatory compliance of newly emerging aqueous-based FAV formulations, such as oral solutions and inhalation therapies. In alignment with green analytical chemistry (GAC) principles, the method’s sustainability was critically assessed using contemporary evaluation tools to demonstrate its suitability for routine application in environmentally conscious laboratories.

## Experimental

### Instrumentation

All the measurements were recorded using a SHIMADZU RF-6000 spectrofluorometer (Tokyo, Japan). The fluorometer was equipped with a 150 W xenon lamp and dual monochromators for excitation and emission wavelength from 200 to 600 nm. The bandwidth was set to 10.0 nm, with a scan speed of 6000 nm/min and auto sensitivity. The instrument was controlled by LabSolution RF software V1.01. A JENWAY pH meter was used for pH adjustment.

### Materials and reagents

During the experiment, only analytical-grade chemicals and reagents were used. Purchases from PIOCHEM (Egypt) included hydrochloric acid and organic solvents such as acetone, methanol, ethanol, n-hexane, and propylene glycol. Polyvinyl alcohol and poloxamer 407, two surfactants, were bought from Sigma-Aldrich (Germany), while sodium hydroxide was acquired from VWR Chemicals (US).

Favipiravir (100.03 ± 0.61%) was kindly supplied by Marcyrl Pharmaceutical Industries (Egypt). Pirafavi^®^ tablets manufactured by Marcyrl Pharmaceutical Industries (Egypt), batch no. 2,132,642, were purchased from local market. Each tablet is labeled to contain 200 mg of FAV.

### Solutions

#### Preparation of FAV standard and working stock solutions

The FAV stock solution, with a concentration of 100 µg/mL, was prepared in distilled water. An accurately weighed amount of 10 mg FAV was dissolved in 40 mL water in a 100-mL volumetric flask, sonicated for 5 min, and then the volume was diluted to volume with distilled water.

A working standard solution containing 100 ng/mL of FAV was prepared by transferring 100 µL from the standard stock solution to a 100-mL volumetric flask, the volume was then diluted to volume with distilled water.

#### Preparation of acid-induced degradation product (ADP) stock and working solutions

According to previous literature [21, 29, 38], the preparation of ADP was performed. Briefly, 25 mg of FAV was dissolved in 25 mL of 1.0 N HCl and refluxed at 100 °C for 2 h. After cooling, the solution was neutralized with 1.0 N NaOH and diluted with water to a final volume of 100 mL in a volumetric flask, resulting in a concentration of 250 µg/mL. After that an aliquot of 4 mL was transferred to a 10-mL volumetric flask, and the volume was diluted to volume with distilled water to create a stock solution (100 µg/mL). A suitable aliquot was withdrawn into a 100-mL volumetric flask, and the volume was diluted to volume with distilled water to produce a working solution (100 ng/mL).

### Procedure

#### Spectral characteristics

The excitation and emission spectra of 50 ng/mL FAV and ADP solutions were recorded against water as a blank over the range of 200–600 nm. The emission spectra were recorded after excitation at λ_ex_ 364.6 nm.

#### Construction of calibration curve

Aliquots equivalent to 50–800 ng FAV were accurately transferred from the working standard solution (100 ng/mL) into a series of 10-mL volumetric flasks, sonicated for 20 min, and the volume was diluted to volume with distilled water to obtain a concentration range of 5–80 ng/mL. The prepared solutions were cooled to 8 °C in a refrigerator, and their fluorescence spectra were then recorded over the range of 365–600 nm. D^0^ spectra were manipulated to obtain their D^1^ spectra with a scaling factor of 10 and Δλ = 4. A calibration curve was constructed from the D^1^ spectra by plotting FAV peak amplitudes at 416.5 nm versus its corresponding concentrations, and the regression equation was calculated.

#### Application to laboratory-prepared mixtures

Laboratory-prepared mixtures were prepared in various ratios by transferring different aliquots from FAV and ADP working solutions into a series of 10-mL volumetric flasks and completing the volume with distilled water. The fluorescence spectra of these mixtures were then recorded after cooling in a refrigerator. The concentration of FAV was calculated following the steps described under “Construction of calibration curve”.

#### Application to Pirafavi^®^ tablets

Ten tablets of Pirafavi^®^ were weighed, and their contents were ground to a fine powder after removing their coats. A quantity equivalent to 10 mg of FAV was transferred to a 100-mL volumetric flask and dissolved in 30 mL methanol; the solution was sonicated for 5 min, filtered through a 0.45 μm membrane filter, and then the volume was diluted to volume with distilled water. Suitable dilutions were made from the prepared tablets solution (100 µg/mL) to reach a concentration of 50 ng/mL, and fluorescence spectra were recorded after cooling in a refrigerator. The procedure under “Construction of calibration curve” was applied, and the concentrations of FAV were computed using the corresponding regression equation.

## Results and discussion

Previous studies have demonstrated that FAV is prone to hydrolysis, necessitating the preparation of its degradation product prior to stability-indicating method development to support the development of newly emerging aqueous-based FAV formulations [35–37]. The literature further emphasizes FAV’s increased susceptibility to acid hydrolysis relative to other degradation conditions [32–34]. Consequently, forced degradation study was conducted under acidic conditions to evaluate its degradation profile and prepare its potential hydrolytic degradation product. The degradation of FAV and characterization of its ADP via IR and MS were carried out according to previous literature [21, 29, 38]. These degradation experiments, which employ aqueous acid/base solutions, are not required in routine quality-control measurements, where only aqueous FAV samples are analyzed.

The FAV structure contains an amide group that is likely to undergo hydrolysis under acidic conditions. To verify this, FAV and its ADP were analyzed using IR and LC/MS. The IR spectrum of FAV exhibited a forked peak at 3400 cm⁻¹, corresponding to the NH₂ group of the amide (Figure S1a, Supplementary Materials). In contrast, the IR spectrum of ADP lacked this NH₂ peak and instead showed a broad OH stretching band at 3440 cm⁻¹, characteristic of a carboxylic acid (COOH) group (Figure S1b, Supplementary Materials). The mass spectrum of ADP displayed a molecular ion peak at m/z 157, confirming its molecular weight. The mass spectrum of ADP showed a weak molecular ion peak at m/z 157 (M–1), consistent with its molecular weight of 158, as the ESI source was operated in negative ion mode (Figure S2, Supplementary Materials).This finding suggests that FAV undergoes hydrolytic degradation via cleavage of the amide bond, resulting in the formation of a carboxylic acid and ammonium chloride salt [38].

### Optimization of experimentalconditions

**Fig. 1:**
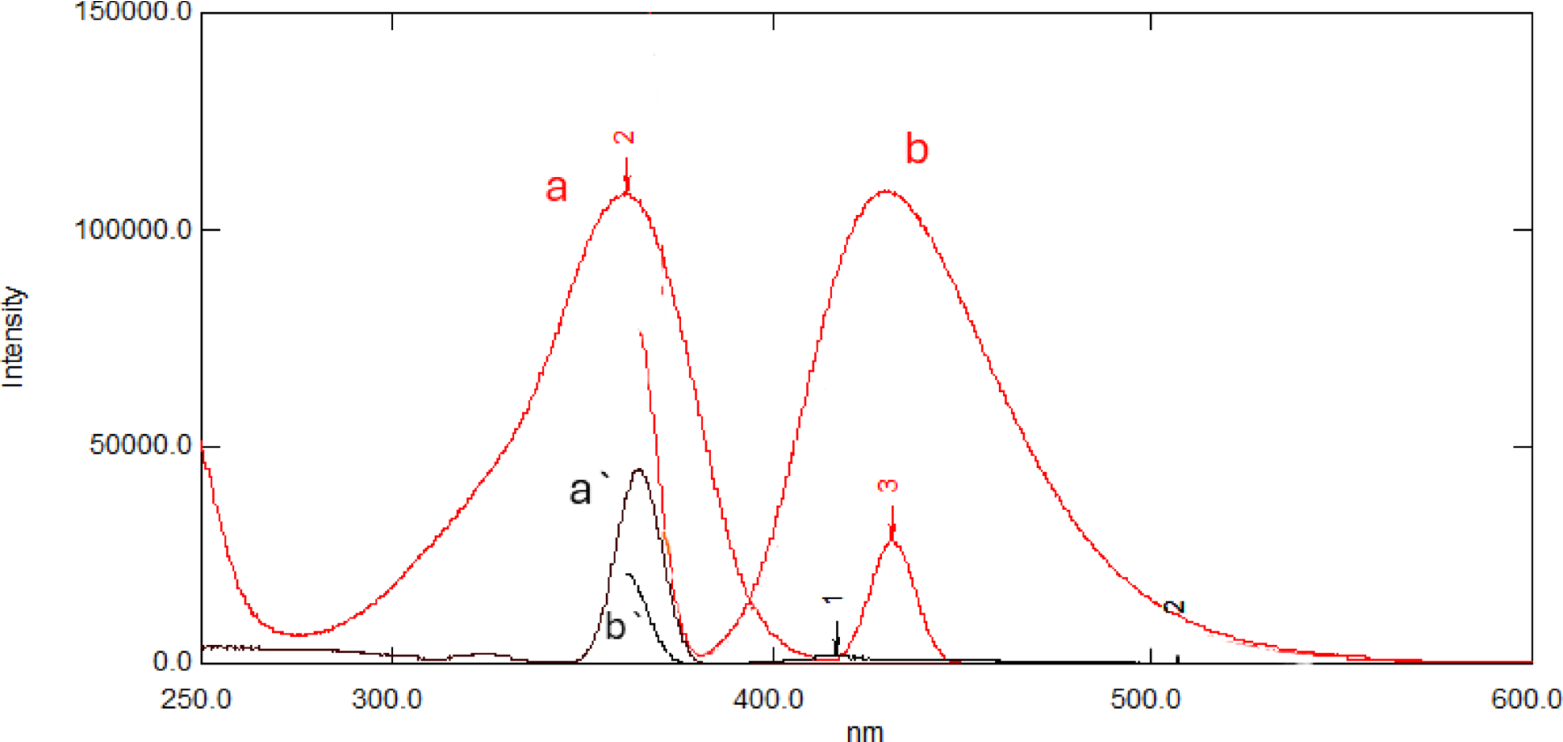
Overlaid fluorescence spectra of Favipiravir (50 ng/mL) and its Acid-Induced Degradation Product (50 ng/mL) in distilled water. (**a**,** a’**) Excitation spectra of Favipiravir (red line) and degradation product (black line). (**b**,** b’**) Emission spectra of Favipiravir (red line) and degradation product (black line)

The excitation and emission spectra of 50 ng/mL of FAV and ADP solutions were recorded against water as a blank over the range of 200–600 nm. Different excitation wavelengths such as 233.4, 360 and 364.6 were tried to obtain the maximum fluorescence intensity for FAV. Therefore, emission spectra were recorded after excitation at λ_ex_ 364.6 nm, at which the highest sensitivity for FAV was obtained (Fig. 1). The variables that influence the sensitivity of FAV fluorescence were optimized including different solvents, pH, surfactant, sonication time, and scan speed were investigated.

**Fig. 2:**
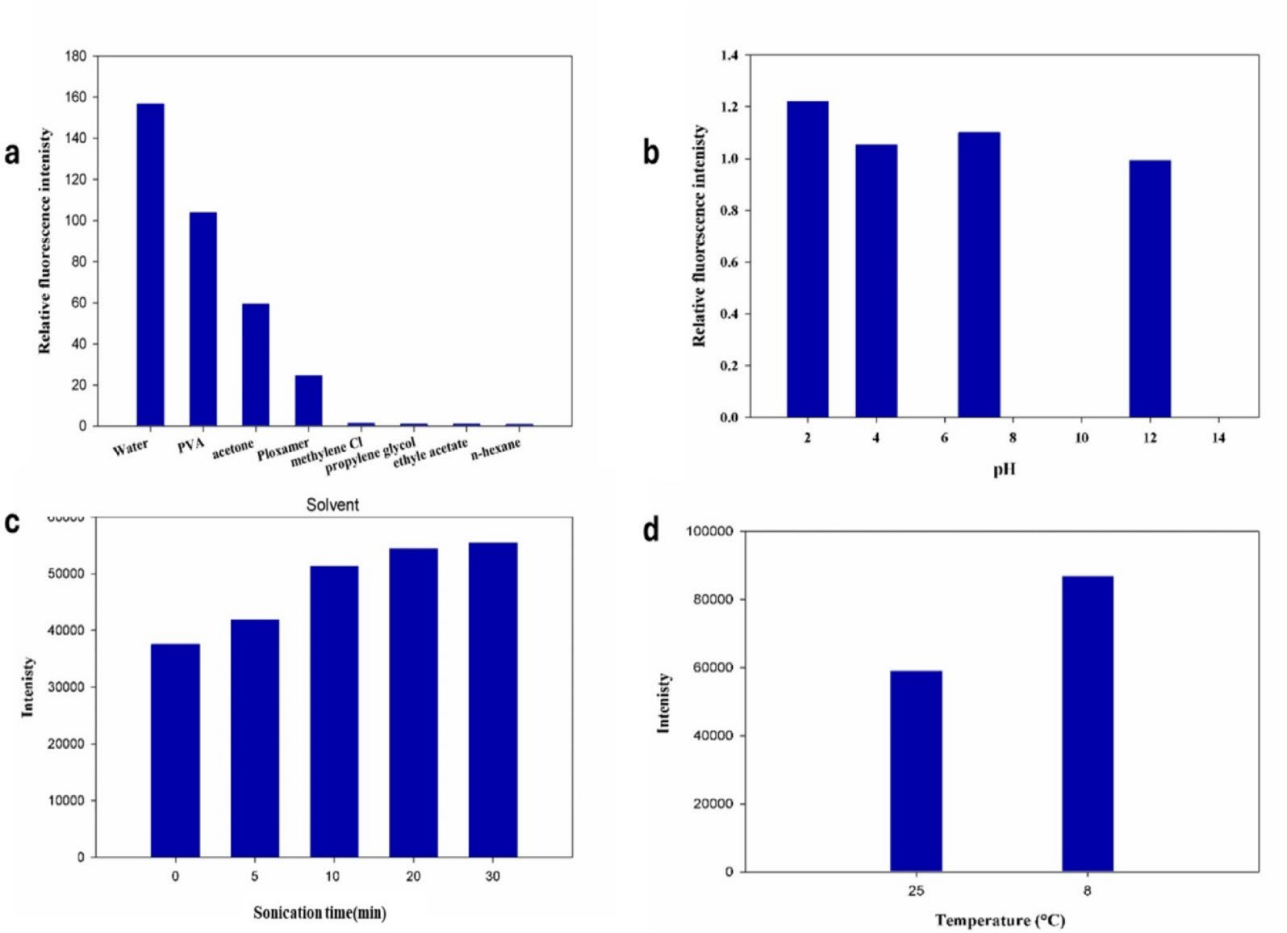
Optimization of Favipiravir (50 ng/mL) Fluorescence, **a** Solvents and surfactants, **b** pH, **c** Sonication, and **d** Temperature

Water, acetone, methylene chloride, propylene glycol, ethyl acetate, and n-hexane were among the solvents tested. It was found that using water produced the maximum intensity of the drug under study compared to other solvents as shown in Fig. 2a. Additionally, the effect of pH on the fluorescence intensity of the studied drug was examined by using buffer solutions, such as Britton–Robinson buffer (BRB), as diluents. These buffers span a wide pH range, allowing for a comprehensive evaluation of pH influence, as illustrated in Fig. 2b. Although fluorescence intensity was high at pH 2, the results showed that the intensities attained were lower than those in pure distilled water. In contrast, sonication time was found to have a significant impact on intensity. Experimentation with sonication times ranging from 5 to 30 min was performed, and 20 min was the optimum time for maximum intensity (Fig. 2c). Studying the effect of temperature at room conditions and after cooling revealed that refrigeration markedly increased the fluorescence intensity, as illustrated in Fig. 2d. Consequently, all measurements were performed at 8 ± 1 °C after cooling in a refrigerator. Conversely, the addition of various surfactants such as poloxamer and polyvinyl alcohol (PVA) did not produce any significant enhancement in fluorescence intensity; therefore, no surfactants were employed in the final procedure (Fig. 2a).

### Development of the spectrofluorimetric method

**Fig. 3:**
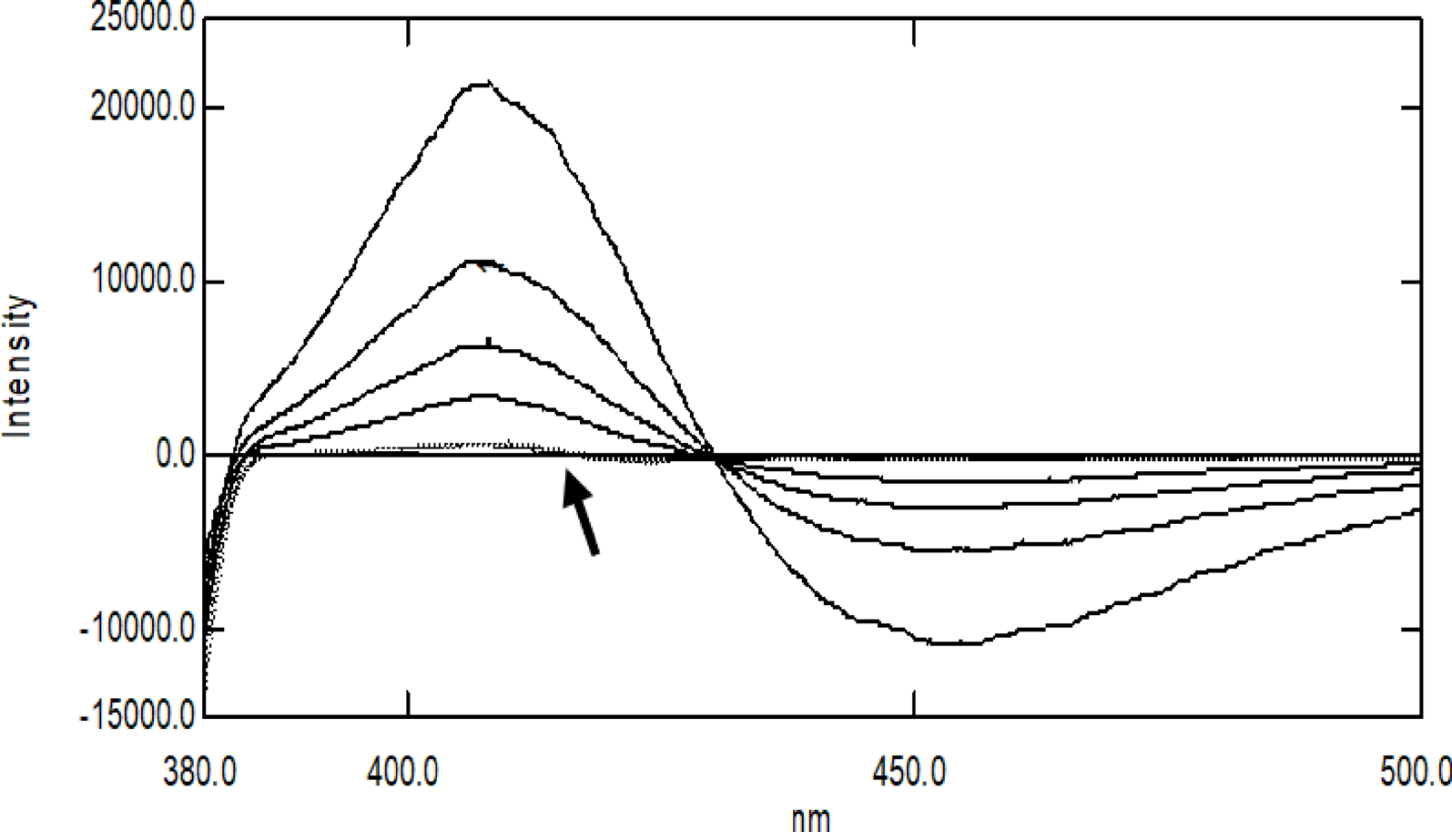
First derivative fluorescence spectra of Favipiravir (–––) and acid-induced degradation product (- - - -) showing a zero crossing point at 416.5 nm for FAV determination

It was challenging to perform a direct assay for FAV in the presence of ADP using a standard fluorescence mode because of the overlap between their emission fluorescence spectra (Fig. 1). Nonetheless, the spectral overlap issue was resolved by mathematically converting zero-order (D^0^) fluorescence spectra to their first-order derivative (D^1^) spectra. Different ∆λ and scaling factors were tried to obtain maximum sensitivity for FAV, and ∆λ = 4 and a scaling factor of 10 were selected as the best conditions for obtaining satisfactory peak resolution and sensitivity. Under the optimized conditions for FAV emission, FAV was determined at 416.5 nm, where a zero-crossing point for ADP was obtained, as shown in Fig. 3.

### Method validation

The method validation was carried out in accordance with ICH guidelines [39]. Table 1 shows the metrics for linearity, accuracy, precision, robustness, LOD, and LOQ.

Good linearity was shown by the developed method over the concentration range of 5–80 ng/mL, where fluorescence intensity was recorded and plotted against concentration using water as the blank. The regression parameters values are listed in Table 1. In addition, LOD and LOQ were calculated as 3.3 σ/S, and 10 σ/S, respectively, where (σ) is the residuals standard deviation and (S) is the slope of the calibration curve. The method showed good sensitivity with LOD and LOQ of 1.6 and 4.8 ng/mL, respectively.

By computing recovery percentage (R%) for five distinct FAV concentrations in triplicate (15, 25, 35, 50, and 75 ng/mL), the developed method’s accuracy was verified by the closeness of calculated R% to the true values, as shown in Table 1. Repeatability (intra-day) and intermediate precision (inter-day) were established at three different measurements (10, 40, and 50 ng/mL) within the same day and on three different days, respectively. Table 1 displays acceptable precision, with RSD% values not exceeding 1.4%.

Specificity of the method to determine FAV in the presence of its ADP was confirmed by analysis of laboratory-prepared mixtures. Mixtures with varying proportions of FAV and ADP were prepared, and the emission spectrum of each mixture was recorded. The process was then completed as previously stated. The regression equation was used to calculate FAV concentration and R%. The proposed fluorimetric method showed that FAV could be accurately measured even in the presence of up to 80% ADP, as illustrated in Table 2.

The robustness of the developed method was assessed to confirm and ensure that it can withstand small changes in the experimental conditions. Trials were conducted to determine the effect of minor changes in temperature (8 ± 1 °C ) and sonication time (20 ± 2 min). As shown in Table 1, changes in intensity, measured in terms of RSD%, were found to be within permissible limits. Short-term stability was also studied, and standard FAV solutions showed satisfactory stability for 24 h in refrigrator (Table 1).

**Table 1:** Validation parameters data of the proposed D^1^ fluorometric method for favipiravir determination in presence of its acid-induced degradation product

Parameter	D^1^
Linearity range (ng/mL)	5–80
Slope	387.5
Intercept	298.6
Regression coefficient (r)	0.9998
Accuracy (mean ± SD) ^a^	99.6 ± 0.9
LOD (ng/mL) ^b^	1.6
LOQ (ng/mL) ^b^	4.8
Intra-day precision ^c^	1.1
Inter-day precision ^d^	1.4
Temp (8 ± 1 °C) ^e^	0.7
Sonication time (20 ± 2 min) ^e^	1.2
Stability (24 h) ^e^	0.6

^a^Mean recovery percentage (*n* = 3) of five concentrations (15, 25, 35, 50, and 75 ng/mL)

^b^Limits of detection “LOD” and quantitation “LOQ” are determined via calculations, LOD = 3.3 * SD of the residuals/slope, LOQ = 10 * SD of the residuals/slope

^c^RSD% of three concentrations (10, 40, 50 ng/mL) repeated three times within the same day

^d^RSD% of three concentrations (10, 40, 50 ng/mL) repeated three times in three successive days

^e^RSD% for the fluorescence intensity at different conditions

**Table 2:** Determination of favipiravir in laboratory-prepared mixtures with its acid-induced degradation product (ADP) with the proposed D^1^ fluorimetric method

Concentration (ng/mL)		D^1^
FAV	ADP	Recovery % ^a^
36	**4 (10%)**	98.7
32	**8 (20%)**	99.1
28	**12 (30%)**	101.4
24	**16 (40%)**	98.7
20	**20 (50%)**	100.4
8	**32 (80%)**	98.8

^a^ Average of three determinations

### Application to pharmaceutical dosage forms and statistical analysis

The developed fluorimetric method was used to determine the FAV content of Pirafavi^®^ tablets. The calculated R% of FAV was compared statistically to the results obtained by the reported method [35]. Student’s t-test and F-test results showed no significant difference, demonstrating that the proposed method can be successfully applied for analysis of FAV dosage forms in QC laboratories, as shown in Table 3.

**Table 3:** Statistical analysis of the proposed fluorimetric and reported HPLC methods for the analysis of Pirafavi^®^ tablets

Parameters	Reported method [35]^a^	Fluorimetric method
Mean recovery % ^b^	99.7	98.7
Variance	1.2	0.7
n	4	4
Student’s t-test ^c^	-	1.48 (2.45) ^**c**^
F-test ^c^	-	1.66 (9.28) ^**c**^

^a^Inertsil ODS-3 V C18 maintained at 30 °C, phosphate buffer (pH 3.5) and acetonitrile, (90:10, v/v) as mobile phase at a flow rate of 1.0 mL/min and detection at 358.0 nm

^b^Average of four determinations

^c^Values between the parentheses are the tabulated t- and F- values (*p* = 0.05)

### Method comparison

The proposed spectrofluorimetric method was critically evaluated against all previously reported fluorimetric procedures for FAV determination, as summarized in Table S1 (Supplementary Materials). Reported spectrofluorimetric methods have mainly quantified FAV either alone or in combination with other antivirals or co-administered drugs. However, none of these methods were designed to be stability-indicating, that is, capable of quantifying the drug in the presence of its degradation products. In contrast, the present method was intentionally developed and validated to perform this stability-indicating function, making it directly applicable to the quality control of aqueous FAV formulations, where hydrolytic degradation represents the most relevant pathway. Another distinguishing advantage is its aqueous nature, as all calibration and fluorimetric measurement steps are performed in distilled water without the use of organic solvents or buffer solutions.

In terms of analytical performance, the proposed method exhibits superior sensitivity and linearity (5–80 ng/mL) compared with the previously reported aqueous-based method by Sri et al. [22], as well as the buffer- or solvent-based procedures of Megahed et al. [23], Ramzy et al. [24], and Batubara et al. [26]. Although the synchronous spectrofluorimetric methods of El Sharkasy et al. [25] and El Sherbiny et al. [27] achieved lower LOQ values, their use of organic solvents and buffer systems inherently compromises sustainability due to increased chemical consumption and pH control requirements. Hence, the present method attains an optimal balance between sensitivity, applicability, and environmental performance, offering the first purely aqueous and environmentally benign stability-indicating spectrofluorimetric method for the pharmaceutical quality control of FAV.

On the other hand,chromatographic methods are indeed capable of providing stability-indicating determinations for FAV [33–36]; however, they typically rely on large volumes of organic solvents and require energy-intensive instrumentation (e.g., HPLC or LC–MS/MS), which markedly reduces their greenness and cost-efficiency. Moreover, their working ranges are usually expressed at higher concentration levels (µg/mL scale), limiting sensitivity compared with the proposed fluorimetric procedure. These sustainability and performance aspects are comparatively evaluated in the following section.

### Sustainability assessment

Green analytical chemistry (GAC) is an evolving field focused on developing analytical methods that reduce or eliminate the adverse effects of organic solvents on both human health and the environment [40]. In recent years, with regulatory frameworks emphasizing on the importance of life-cycle management of analytical methods, the principles of GAC have been increasingly applied across various analytical techniques, including spectroscopy [41–44], electrochemistry [45, 46], and chromatography [38, 47]. Fluorimetric methods, in particular, present a sustainable alternative due to their lower operational costs, ease of use, and reduced environmental impact compared to chromatography [48, 49]. These methods enable the utilization of eco-friendly solvents, further enhancing their suitability for green analytical applications in the field of quality control.

The environmental and sustainability characteristics of the proposed spectrofluorimetric method were examined using three comprehensive evaluation tools: GAPI, AGREE, and RGB12. A comparison was conducted with both the reported HPLC method [35] and the two single-component spectrofluorimetric methods—Sri et al. [22] and Megahed et al. [23]—to highlight differences in solvent/reagent use, sensitivity, application scope, and overall greenness (Table 4).

The first tool was the Green Analytical Procedure Index (GAPI), which evaluates the environmental impact of each procedural step using a color-coded pictogram [50]. As shown in Table 4, the GAPI diagram for the proposed spectrofluorimetric method reveals mainly green and yellow areas, reflecting favorable environmental performance. However, the presence of red zones indicates some limitations, such as offline sample preparation and lack of waste treatment, which are common limitations in many manual methods. In contrast, the HPLC pictogram displays several red and yellow segments corresponding to the extensive use of acetonitrile and phosphate buffer, indicating higher toxicity and waste generation. The Megahed method, though greener than HPLC due to using only a borate buffer system, still introduces a mildly irritant reagent. The Sri method, which also used water as a solvent, shows a GAPI pattern nearly identical to ours but lacks the stability-indicating capability and sensitivity of the proposed procedure. Thus, while Sri’s approach matches the proposed method in terms of solvent greenness, the current work achieves superior performance without sacrificing environmental compliance.

The second evaluation tool was the AGREE metric, which visually represents adherence to the 12 principles of GAC in a circular diagram. Each segment ranges in color from red (score of 0) to green (score of 1), with an overall greenness score displayed at the center [51]. The proposed method achieved an AGREE score of 0.88, as illustrated in Table 4, indicating a high level of compliance with GAC principles. By comparison, the HPLC method scored 0.66, the Megahed method 0.73, and the Sri method 0.86. The differences are most evident in sections related to solvent hazards and energy consumption (Sects. 9, 10, and 11): HPLC shows red coloration due to corrosive, flammable reagents and high power demand; Megahed’s score is penalized by buffer preparation and pH control; whereas the Sri and proposed methods show nearly full green coverage, reflecting solvent-free operation and minimal energy input.

The third tool used was the Whiteness (RGB12) metric, which evaluates the overall sustainability of an analytical procedure. It integrates analytical performance (red), environmental impact, and cost/practicality (blue) into a single composite “whiteness” score [52]. The proposed method exhibited the highest overall RGB12 score of 98.7%, surpassing HPLC (89.3%), Megahed (95.9%), and Sri (96.5%). While the Sri method showed similar greenness (green and blue components), the red analytical-performance domain distinguishes the present method through its lower LOQ and stability-indicating nature. Likewise, Megahed’s slightly lower score reflects its dependence on buffer media and reduced sensitivity relative to the solvent-free procedure developed herein. The HPLC method again recorded the lowest score, reflecting its heavy solvent use, high energy demand, and limited analytical sensitivity.

It is worth noting that while the proposed routine fluorimetric method is highly eco-friendly, the preliminary forced degradation study, required to validate the method’s stability-indicating capability, involved standard chemical degradation pathways (acid hydrolysis). These conditions were excluded from the sustainability assessment, as this is a one-time validation step that is not part of the routine quality control procedure.”

In conclusion, based on a side-by-side comparison using GAPI, AGREE, and RGB12 tools, the proposed spectrofluorimetric method demonstrates a superior environmental profile, higher greenness scores, and enhanced sustainability when compared to the reported HPLC and earlier fluorimetric methods. The HPLC technique ranks lowest in greenness and sensitivity, owing to its reliance on organic solvents, greater waste output, and higher operating costs. Meanwhile, the Sri and Megahed methods approach the proposed procedure in ecological terms but fall short in analytical performance. Collectively, these findings confirm that the present aqueous-based, eco-friendly, and stability-indicating spectrofluorimetric method provides the most sustainable and analytically robust option for the determination of FAV, particularly suited for routine quality control of emerging aqueous oral and inhalation formulations.

**Table 4:** Comparison between the proposed spectrofluorimetric method and reported HPLC and spectrofluorimetric methods for determination of favipiravir

	Proposed method	Sri et al. method [22]	Megahed et al. method [23]	HPLC method [35]
Technique	Spectrofluorimetry	Spectrofluorimetry	Spectrofluorimetry	HPLC
Solvents	Water	Water	0.2 M borate buffer pH 8.0	Phosphate buffer pH 3.5 and acetonitrile (90:10, v/v)
Range (ng/mL)	5–80	2000–10,000	40–280	50,000–250,000
Application	Stability-indicating in dosage form	Dosage form	Dosage form and spiked plasma	Stability-indicating in dosage form
AGREE				
GAPI				
RGB 12				

## Conclusion

In this study, a simple, sensitive, and environmentally sustainable spectrofluorimetric method was successfully developed and validated for the quantitative determination of FAV in the presence of its ADP. The proposed method resolves the significant spectral overlap between FAV and its ADP using first-derivative fluorescence spectrometry, enabling selective, accurate, and interference-free quantification. The method exhibits excellent sensitivity, precision, and robustness, and is capable of determining FAV even in the presence of up to 80% of its degradation product. Its successful application to commercial tablet formulations further demonstrates its suitability for routine quality control. Compared with other spectrofluorimetric methods for FAV determination, the present work uniquely combines aqueous analytical determination, stability-indicating capability, and superior analytical performance, achieving an optimal balance between sensitivity, applicability, and environmental sustainability. In light of the ongoing development of aqueous-based FAV formulations, this method offers a cost-effective, rapid, and eco-friendly alternative to conventional chromatographic techniques for stability assessment. Its high greenness and whiteness scores, as confirmed by AGREE, GAPI, and RGB12 metrics, affirm its compliance with modern regulatory expectations for sustainable analytical methods. Overall, this work introduces a practically valuable and scientifically novel approach that supports both pharmaceutical innovation and environmental stewardship in the analytical evaluation of FAV.

## Supplementary Information

Supplementary Material 1.

## Data Availability

Data will be made available on request.
