# Supplementary material for: A sensitive, aqueous-based spectrofluorimetric approach for the determination of favipiravir in presence of its acid-induced degradation product
Source: BMC Chem. 2026 Feb 10;20(1):56. doi: 10.1186/s13065-026-01724-1 (PMC12990654; doi:10.1186/s13065-026-01724-1)
Supplement: Supplementary file 1 — Supplementary Material 1. [file 13065_2026_1724_MOESM1_ESM.pdf]

Supplementary Materials for

A Sensitive, Aqueous-Based Spectrofluorimetric Approach for Determination of Favipiravir in presence of Its Acid-Induced Degradation Product

Mai H. Abd El-Fattah [a,*], Yasmine A. Sharaf [b], Heba M. El-Sayed [b], Said A. Hassan [c,*]

[a] Pharmaceutical Analytical Chemistry Department, College of Pharmaceutical Sciences and Drug Manufacturing, Misr University for Science & Technology, 6th of October City, Giza 12566, Egypt

[b] Analytical Chemistry Department, Faculty of Pharmacy, Zagazig University, Zagazig 44519, Egypt

[c] Pharmaceutical Analytical Chemistry Department, Faculty of Pharmacy, Cairo University, Cairo 11562, Egypt

***Corresponding authors:**

Mai H. Abd El-Fattah

E-mail: mai.hisham@must.edu.eg

Tel.: +201069154440

Said A. Hassan

E-mail: said.hassan@pharma.cu.edu.eg

Tel.: +201000994542

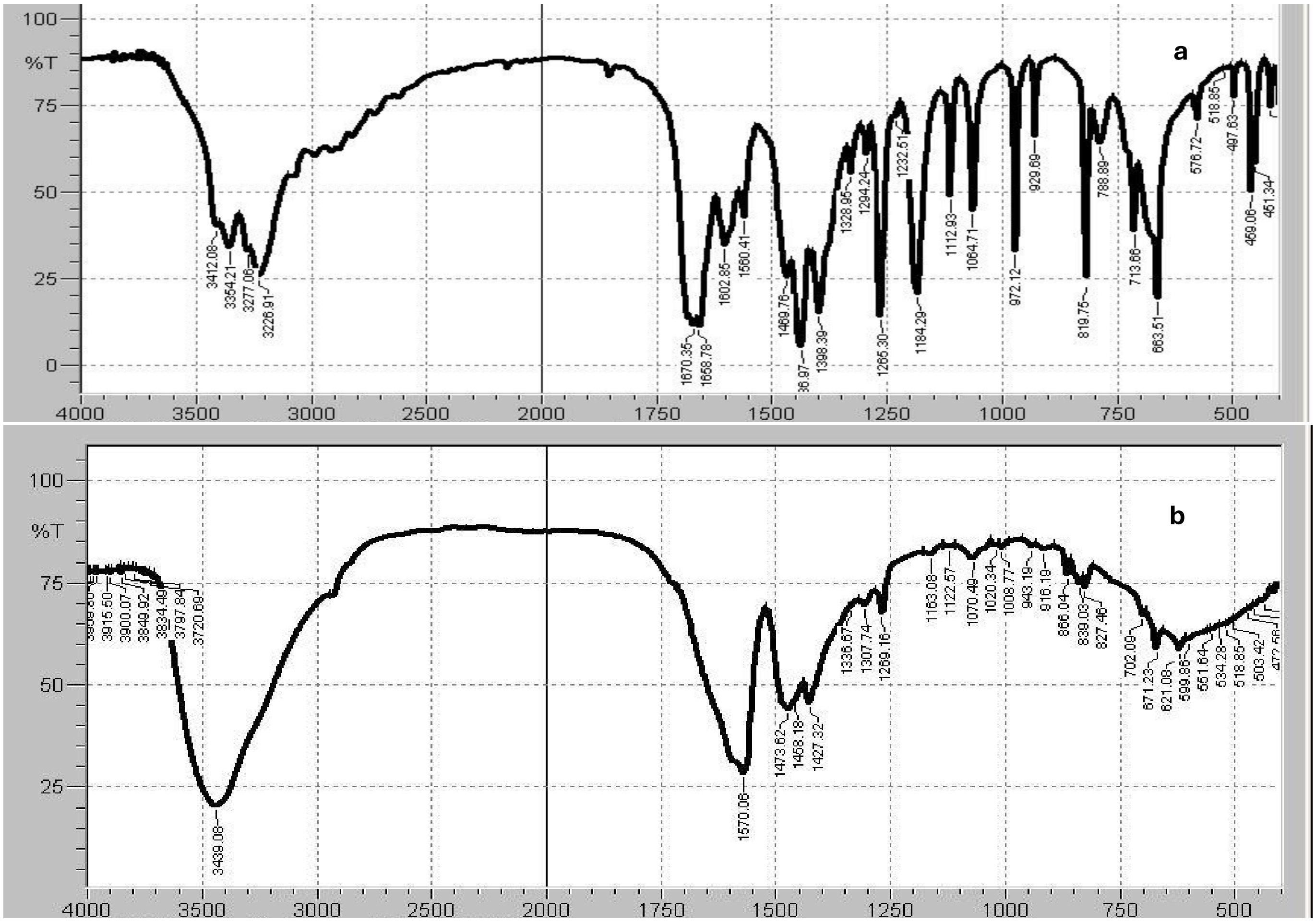

Figure S1. IR spectra of a) Favipiravir (FAV) and b) acidic degradation product (ADP).

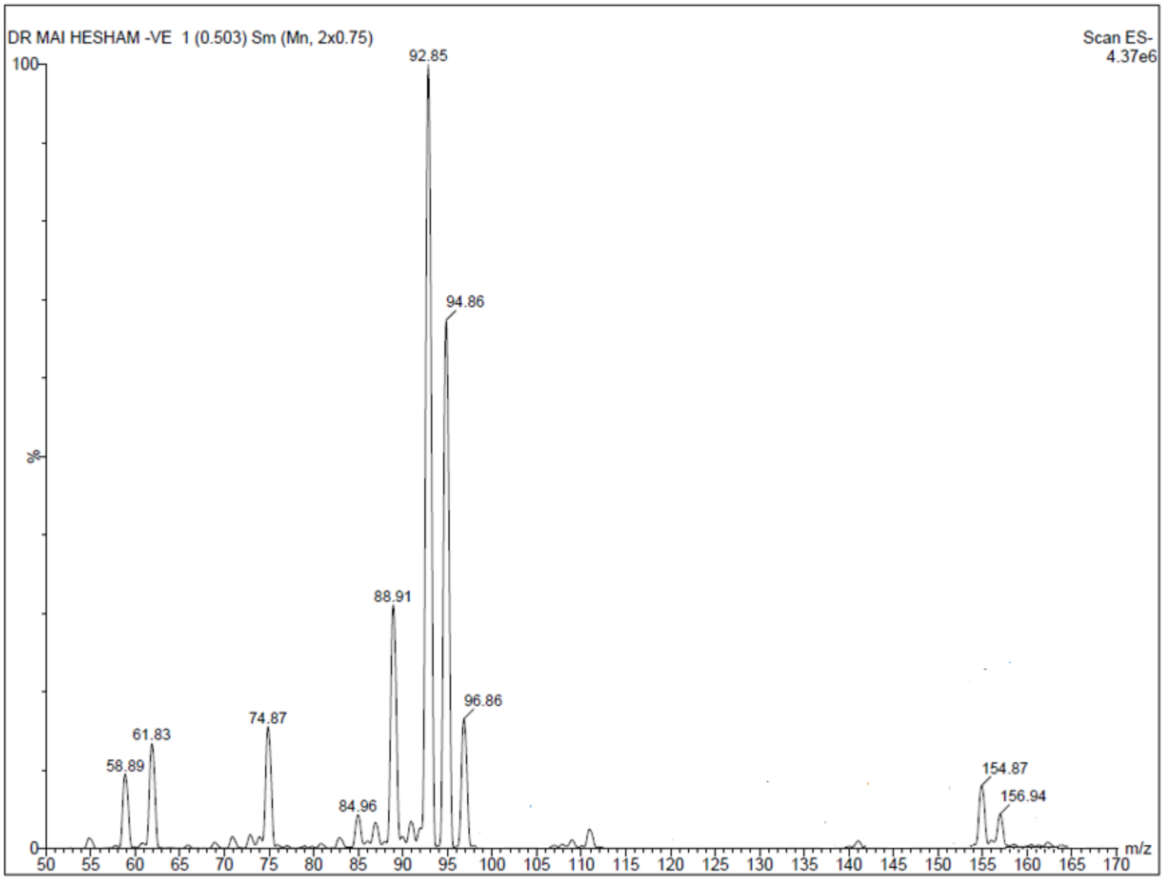

Figure S2. Mass spectrum of acidic degradation product (ADP) of Favipiravir.

Table S1. Comparison between the proposed aqueous spectrofluorimetric method and previously reported spectrofluorimetric methods for favipiravir (FAV) determination.

Study	Analytes	Medium	FAV range (ng/mL)	Stability-indicating method
Proposed Method	**FAV + degradation product**	**Water**	5–80	**Applicable**
Sri et al. [1]	FAV	**Water**	2000–10000	NA
Megahed et al. [2]	FAV	Borate buffer pH 8	40–280	NA
El Sharkasy et al. [3]	FAV + Hydroxychloroquine	Ethanol<br>Acetate buffer pH 5.4	**1–18**	NA
Ramzy et al. [4]	FAV + Remdesivir + Hydroxychloroquine	Ethanol<br>Acetate buffer pH 4	10–400	NA
Batubara et al. [5]	FAV + Aspirin	Ethanol<br>Acetate buffer pH 4	10–500	NA
El Sherbiny et al. [6]	FAV + Molnupiravir	Borate buffer pH 8	2–13	NA

* Shaded cells indicate criteria where the method demonstrates superior performance relative to the other methods.

** NA = Not applicable.
